# Learning styles of medical students and related factors

**DOI:** 10.1186/s12909-023-04267-4

**Published:** 2023-04-25

**Authors:** Albena Gayef, Ayşe Çaylan, Selahattin Alp Temiz

**Affiliations:** 1 Medical School, Department of Medical Education, Trakya University, Edirne, Türkiye; 2 Medical School, Department of Family Medicine, Trakya University, Edirne, Türkiye; 3 Student of Medical School, Trakya University, Edirne, Türkiye

**Keywords:** Learning, Learning style, Medical, Student

## Abstract

**Background:**

The concept of learning style is quite important for teachers to teach, organize students’ learning experiences, and accomplish educational goals. Motivation is one of the most important psychological concepts in education. Motivation is multidimensional and ranges from amotivation to extrinsic motivation and intrinsic motivation. When students are motivated extrinsically, they enjoy striving toward rewards and goals which may differ from individual goals. Intrinsically motivated students enjoy exploring, learning, and curiosity-oriented academic efforts. Understanding learning styles can make it easier to create, modify, and develop more efficient curriculum and educational programs. It can also encourage students’ participation in these programs and motivate them to gain professional knowledge This study aims to determine the learning styles of medical school students and to evaluate whether there is a relationship between their learning styles and academic motivation and the sociodemographic variables.

**Methods:**

In this study a questionnaire containing socio-demographic factors, Grasha-Reichmann Learning Styles Scale, Academic Motivation Scale was filled out by 1st, 2nd, 3rd, 4th, and 5th -year medical students of the 2019–2020 academic year. Frequency, percentage, mean, ANOVA, Pearson correlation analysis, and independent group t-test (for analyzing data with normal distribution) were applied. Mann Whitney U test, Kruskal Wallis test, and Spearman correlation analysis were used for analyzing data without normal distribution.

**Results:**

We found that the mean of independent learning was the highest among the learning style dimensions, and the mean of the intrinsic motivation to know (IMKN) was the highest among the academic motivation dimensions. We found that there were significant relationships between independent learning and intrinsic motivation (IM), between avoidant learning and extrinsic motivation (EM) and between collaborative learning and IMKN, IM to accomplish things (IMAT) and IM to experience stimulation (IMES).

**Conclusion:**

We think that different teaching methods can be applied to strengthen collaborative learning, participant learning, and intrinsic motivation. We hope that this research will contribute to medical education on the subject of establishing appropriate teaching methods. Teachers have to plan and implement activities based on students’ learning styles and academic motivation to encourage students to effectively participate in the classroom.

**Supplementary Information:**

The online version contains supplementary material available at 10.1186/s12909-023-04267-4.

## Background

Learning styles and teaching styles play an important role in medical education. Shaping education according to learning styles in students with low motivation will increase their academic motivation and academic motivation is important to enhance learning. The concept of learning style, which refers to the students’ preferences in the learning process and learning conditions, is quite important for teachers to teach, organize students’ learning experiences, and accomplish educational goals.

Grasha-Reichmann defined learning styles as independent, avoidant, collaborative, dependent, competitive and participant [1]. According to Grasha (2002), competitive learners like to be the center of attention and receive recognition, collaborative learners can work together with teachers and others in the classroom, avoidant learners are uninterested and overwhelmed by things around them, participant learners enjoy going to class and taking part in classroom activities, dependent learners are the ones who need guidance and instruction from teachers or peers on things they have to do, and independent learners are the ones prefer to learn the content themselves. No learning style is better or worse than any other. Each person has different learning styles based on their unique abilities [2]. Learning styles are factors that directly affect students’ learning process; the understanding of those factors allows teachers to develop appropriate teaching methods to improve students’ performance [3]. Learning style alone is not the only factor that may influence a learning situation. Students’ learning preferences may be influenced by several factors, including gender, age, major, sociocultural factors, educational and cultural context of university, individual awareness, life experience, other learning skills, effect of educator, motivation, amount of academic assistance received and teaching style of mentor etc. [4–6]. Contradictory results regarding these potential influences have been reported in various studies. It is important to identify students’ learning style preferences in order to design an effective educational curriculum [5].Studies about learning show that considering learning styles in planning and presenting education can improve learning processes meaningfully [7]. In particular, the programs prepared according to the recent constructivist approach have revealed that a different momentum will be gained by identifying the students’ learning styles, characteristic features, individual differences, and student-centered understanding. The individual characteristics, educational understanding, and the methods/techniques that the teachers will consider during teaching also differ. Determining the methods and techniques to be used to get the desired level of product in education is an essential point in the learning/teaching process. In this process, it is important to identify the individuals’ learning styles by considering their differences, readiness levels, interests, and genetic characteristics and to provide education in line with this [8].

Motivation is one of the most important psychological concepts in education. Motivation is multidimensional and ranges from amotivation (AMO) to extrinsic motivation (EM) and intrinsic motivation (IM) [9]. External motivation arises by external factors such as family, teachers and environment. External motivation can be given in the form of praise, gifts, good grades, incentives and so on. On the other hand, intrinsic motivation is encouragement for a person to do something for his/ her self- interest and satisfaction. Intrinsic motivation differs based on students’ characteristics. IM or EM is an essential factor for ensuring students’ participation in learning activities in their environment. IM is the urge to pursue an activity solely for pleasure and satisfaction. EM is the urge to pursue an activity without obligation or as a means to achieve a goal. AMO, on the other hand, is the lack of intention or urge to perform an activity because of the inability to establish a possible relationship between behavior and activity [10].

Deci and Ryan (2000) divided EM into four groups: EM-External Regulation (EM-ER), EM-Introjection (EM-IJ), EM-Identification (EM-ID), and EM-Integration (EM-IG) [11]. These four different levels of EM differ according to the degree of autonomy in connection with where the individual’s behavior is more internalized or more integrated. Motivation is more internalized moving from EM-ER to EM-IG [12]. In their study, Vallerand et al. (1992) divided IM into three [13]: IM to know (IMKN), IM to accomplish things (IMAT) and IM to experience stimulation (IMES). IMKN is the individual’s willingness to engage in an activity for the pleasure of learning new things. IMAT is the individual’s willingness to engage in an activity for the satisfaction of accomplishing new things. IMES, on the other hand, is the individual’s willingness to engage in an activity for the satisfaction received while acting and moving [11]. Students need to guide themselves to participate in learning activities in their environment and to develop and use new motivational strategies [14].

The study, conducted by Rashid (2007) stated that there is a significant relationship between students’ learning styles and intrinsic motivation. Learning style which is consistent with students’ motivation allows a student to explore his/ her potentials and capabilities. In another study students’ learning styles are linked tightly either to intrinsic or extrinsic motivation [2]. Parents with higher educational levels tend to give higher premium to education. One’s motivation is highly determined by the cognitions and beliefs toward situations or events and by the outcome of the both personal and interpersonal interactions with the environment [6].

The primary aim of teaching is to facilitate the learning process. Understanding the learning behavior of students is considered to be a part of this process. Research has shown that individuals exhibit different approaches in the learning process and a single strategy or approach was unable to provide optimal learning conditions for all individuals. This may be related to students’different backgrounds, strengths, weaknesses, interests, ambitions, levels of motivation, and approaches to studying. Learning styles may be useful to help students and educators understand how to improve the way they learn and teach, respectively. Determining students’ learning styles provides information about their specific preferences. Understanding learning styles can make it easier to create, modify, and develop more efficient curriculum and educational programs. It can also encourage students’ participation in these programs and motivate them to gain professional knowledge [4].

This study aims to determine the learning styles of medical school students and to evaluate whether there is a relationship between their learning styles and academic motivation and the socio-demographic variables. For this study our research questions and hypothesis were as follows:

Research Questions:

1. What is the relationship between learning styles and sociodemographic variables of medical students?
2. What is the relationship between academic motivation and sociodemographic variables of medical students?
3. What is the relationship between learning styles and academic motivation of medical students?

Research Hypothesis:

### Ho1

There is no significant relationship between learning styles and sociodemographic variables of medical students.

### Ho2

There is no significant relationship between academic motivation and sociodemographic variables of medical students.

### Ho3

There is no significant relationship between learning styles and academic motivation of medical students.

## Methods

This study is a cross-sectional study. In this study a questionnaire containing socio-demographic factors, Grasha-Reichmann Learning Styles Scale, Academic Motivation Scale was filled out by 1st, 2nd, 3rd, 4th, and 5th -year medical students of the 2019–2020 academic year. Although there are many scales related to learning styles, we preferred the Grasha-Reichmann Learning Style Scale because there was another study going on about teaching styles of the educators of the same group. Since Grasha-Reichmann had scales on teaching as well as on learning styles we had to use Grasha-Reichmann Learning Styles Scale. Since we aimed to reach the entire universe (N = 1365) only 882 students participated in the study.

The Grasha-Reichmann Learning Style Scale was adapted to Turkish by Sarıtaş and Süral [8] who performed its validity and reliability study. As a result of the Pearson Correlation Test, they calculated the language validity correlation of the scale and found as 0.62. They applied Cronbach’s Alpha Reliability Test to all data to measure the reliability of the measurement tool, and they also performed an item analysis. The reliability coefficient of the scale was 0.802. Grasha-Reichmann Learning Style Scale consists of 60 five-point Likert-type items with six dimensions (independent, avoidant, collaborative, dependent, competitive, participant) with 10 items per dimension [8].

Academic Motivation Scale was developed by Vallerand et al. in 1992 [13]. The university form of the scale was adapted to Turkish by Karagüven [14] who performed its validity and reliability study. The scale has 28 items. It has seven dimensions (three IM; three EM; one AMO) with four items per dimension. They are IMKN, IMAT, IMES, EM-ER, EM-IJ, EM-ID, and AMO. Scores from subscales range from 4 to 28. Items are evaluated separately so if the score for an item is close to 28, this indicates that this dimension is high for the individual. Each statement can be marked over seven degrees namely between one (not agree at all) and seven (completely agree). Statements in the AMO dimension are reversed compared to the others. However, while scoring, these items are scored like the others. There is no reverse-scored item in the scale. The Cronbach alpha value of the scale was 0.87 [14].

In our study, the Cronbach’s Alpha coefficient of the Learning Styles Scale was 0.80 and the Cronbach’s Alpha coefficient of Academic Motivation Scale was 0.84.

### Statistical analysis

The assumption of normal distribution was checked with histogram and Q-Q plots. In comparisons of two independent groups, t-test or Mann-Whitney U test was used depending on normal distribution. One-way analysis of variance or Kruskall-Wallis test was used for comparisons of more than two independent groups depending on normal distribution. Relationships between numerical variables were investigated with Pearson or Spearman correlation coefficients depending on the normal distribution. The Bonferrroni correction was applied for multiple comparisons. A p-value less than 0.05 was considered statistically significant. Data analyzes were performed with the SPSS 19 (Statistical Package for the Social Sciences, version 19, seri no: 10240462) program.

We received approval from the Scientific Researches Ethics Committee of the Medical School of Trakya University (Decision No: 14/15, Dated: 02.09.2019).

## Results

We found that 57.6% (508) of the students were female; 42.4% (374) of the students were male; 41.6% (367) were first-year students; 12.2% (108) were second year students; 30.4% (268) were third year students; 4.9% (43) were fourth year students; 10.9% (96) were fifth year students. 49.9% (440) of the students had spare some time for studying regularly everyday had regular study habits; 50.1% (442) of the students hadn’t spare some time for studying regularly everyday. 84.1% (742) had no problems in access to internet; 15.9% (140) had problem in access to internet. 71.8% (633) took notes in class; 28.2% (249) hadn’t taking note in class. 98.5% (869) were not working in any job; 1.5% (13) were working in any job. Mother’s educational status was high school for 31.6% (279), bachelor for 31.1% (274), elementary for 25.3% (223), master’s degree for 9.5% (84), PhD for 1.2% (11). Father’s educational status was bachelor for 36.8% (325), high school for 28.1% (248), elementary for 15.8% (139), master’s degree for 14.3% (126), PhD for 3.5% (31). We found that 76.6% (676) had no role model for career choice; 23.4% (206) had a role model for career choice. 91.3% (805) did not go to a private teaching institution; 8.7% (77) went to a private teaching institution. 77.0% (679) of the students’mother doesn’t help while studying, 23.0% (203) of the students’mother helps while studying; 84.9% (749) of the students’ father doesn’t help while studying, 15.1% (133) of the students’ father help while studying.

**Table 1:** Means of learning styles and academic motivation dimensions

Learning style dimensions	Mean ± SS
Independent learning	3.78 ± 0.45
Avoidant learning	2.98 ± 0.61
Collaborative learning	3.60 ± 0.60
Dependent learning	3.53 ± 0.49
Competitive learning	2.76 ± 0.67
Participant learning	3.32 ± 0.58
**Academic motivation dimensions**	
IMKN	5.61 ± 1.15
IMAT	4.58 ± 1.34
IMES	4.42 ± 1.30
EM-ID	5.54 ± 1.21
EM-IJ	4.00 ± 1.36
EM-ER	5.05 ± 1.35
AMO	2.52 ± 1.54

IMKN: Intrinsic motivation to know

IMAT: Intrinsic motivation to accomplish things

IMES: Intrinsic motivation to experience stimulation

EM-ID: Extrinsic motivation identification

EM-IJ: Extrinsic motivation introjection

EM-ER: Extrinsic motivation external regulation

AMO: Amotivation

When we examined the students’ scores, we found that the mean of independent learning was the highest among the learning style dimensions, and the mean of the IMKN was the highest among the academic motivation dimensions (Table 1).

**Table 2:** Relationship between learning styles,academic motivation dimensions, gender

Learning Styles	Gender	Mean ± SD	t	p	p adjusted
Independent learning	Female	3.73 ± 0.44	-4.331	< 0.001	**< 0.001**
	Male	3.86 ± 0.45			
Avoidant learning	Female	2.89 ± 0.60	-4.751	< 0.001	**< 0.001**
	Male	3.09 ± 0.62			
Collaborative learning	Female	3.65 ± 0.56	2.735	0.006	0.078
	Male	3.54 ± 0.65			
Dependent learning	Female	3.58 ± 0.48	3.309	0.001	**0.013**
	Male	3.47 ± 0.50			
Competitive learning	Female	2.69 ± 0.64	− 0.294	0.769	1.000
	Male	2.70 ± 0.71			
Participant learning	Female	3.40 ± 0.57	4.768	< 0.001	**< 0.001**
	Male	3.22 ± 0.58			
**Academic Motivation Dimensions**					
IMAT	Female	4.61 ± 1.32	0.779	0.436	1.000
	Male	4.53 ± 1.36			
IMES	Female	4.49 ± 1.29	1.897	0.058	0.754
	Male	4.32 ± 1.30			
EM-IJ	Female	4.00 ± 1.32	0.025	0.980	1.000
	Male	4.00 ± 1.42			
EM-ER	Female	4.96 ± 1.37	-2.292	0.022	0.286
	Male	5.18 ± 1.33			

IMAT: Intrinsic motivation to accomplish things

IMES: Intrinsic motivation to experience stimulation

EM-IJ: Extrinsic motivation introjection

EM-ER: Extrinsic motivation external regulation

When we compared the learning styles according to gender, we found that males had significantly higher means of independent learning and avoidant learning than females (p < 0.05) (Table 2). On the other hand, females had significantly higher means of dependent learning and participant learning than males (p < 0.05) (Table 2).

**Table 3:** Relationship between academic motivation dimensions and gender

VARIABLES	Gender	N	Mean Rank	Sum of Ranks	Mann Whitney U	Z	p	p adjusted
IMKN	Female	508	467.70	237593.00	81685.000	-3.572	< 0.001	**< 0.001**
	Male	374	405.91	151810.00				
EM-ID	Female	508	467.18	237326.50	81951.500	-3.500	< 0.001	**< 0.001**
	Male	374	406.62	152076.50				
AMO	Female	508	401.67	204048.50	74762.500	-5.457	< 0.001	**< 0.001**
	Male	374	495.60	185354.50				

IMKN: Intrinsic motivation to know

EM-ID: Extrinsic motivation identification

AMO: Amotivation

We also compared the academic motivation dimensions according to gender and found that females had significantly higher means of IMKN and EM-ID than males (p < 0.05) (Table 3), and males had significantly higher means of AMO than females (p < 0.05) (Table 3).

**Table 4:** Relationship between learning styles, academic motivation dimensions, to spare some time for studying regularly everyday

VARIABLES	To spare some time for studying regularly everyday	Mean ± SD	t	p	p adjusted
Independent learning	Yes	3.81 ± 0.44	1.494	0.135	1.000
	No	3.76 ± 0.46			
Avoidant learning	Yes	2.77 ± 0.60	-10.198	< 0.001	**< 0.001**
	No	3.18 ± 0.56			
Collaborative learning	Yes	3.66 ± 0.60	2.540	0.011	0.143
	No	3.55 ± 0.60			
Dependent learning	Yes	3.54 ± 0,51	0.575	0.565	1.000
	No	3.52 ± 0.47			
Competitive learning	Yes	2.78 ± 0.68	3.740	< 0.001	**< 0.001**
	No	2.61 ± 0.65			
Participant learning	Yes	3.48 ± 0.59	8.147	< 0.001	**< 0.001**
	No	3.17 ± 0.53			
IMAT	Yes	4.84 ± 1.26	6.015	< 0.001	**< 0.001**
	No	4.31 ± 1.36			
IMES	Yes	4.57 ± 1.24	3.562	< 0.001	**< 0.001**
	No	4.26 ± 1.34			
EM-IJ	Yes	4.08 ± 1.34	1.908	0.057	0.741
	No	3.91 ± 1.38			
EM- ER	Yes	4.98 ± 1.35	-1.690	0.091	1.000
	No	5.13 ± 1.35			

IMAT: Intrinsic motivation to accomplish things

IMES: Intrinsic motivation to experience stimulation

EM-IJ: Extrinsic motivation introjection

EM-ER: Extrinsic motivation external regulation

The relationship between learning styles, academic motivation dimensions and to spare some time for studying regularly everyday is shown in Table 4.

**Table 5:** Relationship between academic motivation dimensions and to spare some time for studying regularly everyday

VARIABLES	To spare some time for studying regularly everyday	N	Mean Rank	Sum of Ranks	Mann Whitney U	Z	p	p adjusted
IMKN	Yes	440	478.42	210503.00	80997.000	-4.308	< 0.001	**< 0.001**
	No	442	404.75	178900.00				
EM-ID	Yes	440	449.10	197602.50	93897.500	− 0.886	0.375	1.000
	No	442	433.94	191800.50				
AMO	Yes	440	410.08	180437.00	83417.000	-3.685	< 0.001	**< 0.001**
	No	442	472.77	208966.00				

IMKN: Intrinsic motivation to know

EM-ID: Extrinsic motivation identification

AMO: Amotivation

The relationship between academic motivation dimensions and to spare some time for studying regularly everyday is shown in Table 5.

The students who took notes in class had a significantly lower mean of avoidant learning than those who did not (p < 0.05). The students who took notes in class had significantly higher means of dependent learning, participant learning, IMES, IMKN, and EM-ID than those who did not (p < 0.05). The mean of AMO was significantly lower in the students who took notes in class (p < 0.05).

**Table 6:** Relationship between learning styles and years

Learning Styles	Year		Mean Difference	F	p	p adjusted
Independent learning	1.year	2.year	0.003	0.888	1.000	1.000
		3.year	0.048		0.663	1.000
		4.year	0.002		1.000	1.000
		5.year	− 0.043		0.917	1.000
Avoidant learning	1.year	2.year	− 0.230^*^	13.331	0.005	0.065
		3.year	− 0.274^*^		< 0.001	**< 0.001**
		4.year	− 0.297^*^		0.019	0.247
		5.year	− 0.392^*^		< 0.001	**< 0.001**
Collaborative learning	1.year	2.year	0.108	7.031	0.462	**1.000**
		3.year	0.210^*^		< 0.001	**< 0.001**
		4.year	0.336^*^		0.005	0.065
		5.year	0.200^*^		0.030	0.390
Dependent learning	1.year	2.year	0.082	6.273	0.536	**1.000**
		3.year	0.106		0.054	**0.702**
		4.year	0.176		0.166	**1.000**
		5.year	0.259^*^		< 0.001	**< 0.001**
Competetive learning	1.year	2.year	0.155	2.373	0.221	**1.000**
		3.year	0.083		0.535	**1.000**
		4.year	0.190		0.404	**1.000**
		5.year	0.175		0.154	**1.000**
Participant learning	1.year	2.year	0.199^*^	13.963	0.012	0.156
		3.year	0.268^*^		< 0.001	**< 0.001**
		4.year	0.288^*^		0.014	0.182
		5.year	0.378^*^		< 0.001	**< 0.001**

The mean of avoidant learning in 1st year students were significantly lower than that of 3rd -year and 5th -year students (p < 0.05). The collaborative learning means of 1st -year students were significantly higher than that of 3rd -year students (p < 0.05). The dependent learning means of the 1st -year students were significantly higher than that of the 5th -year students (p < 0.05). The participant learning means of the 1st -year students were significantly higher (p < 0.05) than that of the 3rd -year and 5th -year students (p < 0.05) (Table 6).

**Table 7:** Relationship between academic motivation dimensions and years

VARIABLES	Year	N	Mean rank	X^2^	p	p adjusted
IMKN	1.year	367	459.63	8.146	0.086	1.000
	2.year	108	457.63			
	3.year	268	414.78			
	4.year	43	485.52			
	5.year	96	408.94			
EM-ID	1.year	367	470.37	15.350	0.004	0.052
	2.year	108	454.75			
	3.year	268	429.83			
	4.year	43	408.27			
	5.year	96	363.71			
AMO	1.year	367	389.01	58.803	< 0.001	**< 0.001**
	2.year	108	388.29			
	3.year	268	473.81			
	4.year	43	498.12			
	5.year	96	586.48			

IMKN: Intrinsic motivation to know

EM-ID: Extrinsic motivation identification

AMO: Amotivation

We found that the means of AMO differed significantly according to the year (p < 0.05) (Table 7).

- The students who had a role model for career choice had significantly higher means of collaborative learning (p < 0.001), competitive learning (p < 0.001), participant learning (p < 0.001) than those who did not.
- The students who went to a private teaching institution had a significantly higher means of AMO (p < 0.001) than those who did not.
- The students whose fathers helped while studying had significantly higher means of collaborative learning (p < 0.001) and IMAT (p = 0.039) than those who did not.
- There was no significant difference in IMAT, IMES, EM-IJ, EM-ER dimensions according to year, mother’s educational status, and father’s educational status (p > 0.05).
- We found no significant difference in the means of IMKN, EM-ID, and AMO according to the mother’s educational status and father’s educational status (p > 0.05).
- There was no significant difference in learning styles and academic motivation dimensions according to working in an income-generating job, access to internet, mothers helped while studying (p > 0.05).

We found a significant positive correlation between age and avoidant learning; a significant negative correlation between age and collaborative learning; a significant negative correlation between age and competitive learning; a significant negative correlation between age and participant learning; a significant negative correlation between age and IMAT (supplementary file).

There was a significant positive correlation between independent learning and IMAT, IMES; a significant negative correlation between avoidant learning and IMAT, IMES; a significant positive correlation between avoidant learning and EM-IJ and EM-ER. We found a significant positive correlation between collaborative learning and IMAT, IMES and EM-IJ; a significant positive correlation between dependent learning and IMES, EM-IJ, and EM-ER. There was a significant negative correlation between dependent learning and IMAT. There was a significant positive correlation between competitive learning and IMAT, IMES, EM-IJ, EM-ER. In addition, there was a significant positive correlation between participant learning and IMAT, IMES, EM-IJ (supplementary file).

**Table 8:** Relationship between learning styles and academic motivation dimensions

		IMKN	EM-ID	AMO
Independent Learning	r_s_	0.346^**^	0.012	0.036
	p	< 0.001	0.716	0.284
	p adjusted	**< 0.001**	1.000	1.000
	n	882	882	882
Avoidant Learning	r_s_	-0.262^**^	-0.134^**^	0.490^**^
	p	< 0.001	< 0.001	< 0.001
	p adjusted	**< 0.001**	**< 0.001**	**< 0.001**
	n	882	882	882
Collaborative Learning	r_s_	0.392^**^	0.111^**^	-0.097^**^
	p	< 0.001	0.001	0.004
	p adjusted	**< 0.001**	**0.006**	**0.024**
	n	882	882	882
Dependent Learning	r_s_	0.120^**^	0.446^**^	-0.283^**^
	p	< 0.001	< 0.001	< 0.001
	p adjusted	**< 0.001**	**< 0.001**	**< 0.001**
	n	882	882	882
Competetive Learning	r_s_	0.158^**^	0.129^**^	-0.010
	p	< 0.001	< 0.001	0.765
	p adjusted	**< 0.001**	**< 0.001**	1.000
	n	882	882	882
Participant Learning	r_s_	0.328^**^	0.335^**^	-0.429^**^
	p	< 0.001	< 0.001	< 0.001
	p adjusted	**< 0.001**	**< 0.001**	**< 0.001**
	n	882	882	882

**Correlation is significant at the 0.01 level (2-tailed).

* Correlation is significant at the 0.05 level (2-tailed).

IMKN: Intrinsic motivation to know

EM-ID: Extrinsic motivation identification

AMO: Amotivation

We found a significant positive correlation between independent learning and IMKN; a significant positive correlation between avoidant learning and AMO; a significant positive correlation between collaborative learning and IMKN, EM-ID; a significant positive correlation between dependent learning and IMKN, EM-ID; a significant positive correlation between competetive learning and IMKN, EM-ID; a significant positive correlation between participant learning and IMKN, EM-ID; a significant negative correlation between avoidant learning and IMKN, EM-ID; a significant negative correlation between collaborative learning and AMO; a significant negative correlation between dependent learning and AMO; a significant negative correlation between participant learning and AMO (Table 8).

**Table 9:** Relationship between age and academic motivation dimensions

		Age	IMKN	EM-ID	AMO
Age	r_s_	1.0000	-0.114^**^	-0.112^**^	0.219^**^
	p	.	0.001	0.001	< 0.001
	p adjusted	.	**0.013**	**0.013**	**< 0.001**
	n	882	882	882	882
IMKN	r_s_	-0.114^**^	1.000	0.367^**^	-0.280^**^
	p	0.001	.	< 0.001	< 0.001
	p adjusted	**0.013**	.	**< 0.001**	**< 0.001**
	n	882	882	882	882
EM-ID	r_s_	-0.112^**^	0.367^**^	1.000	-0.404^**^
	p	0.001	< 0.001	.	< 0.001
	p adjusted	**0.013**	**< 0.001**	.	**< 0.001**
	n	882	882	882	882
AMO	r_s_	0.219^**^	-0.280^**^	-0.404^**^	1.000
	p	< 0.001	< 0.001	< 0.001	.
	p adjusted	**< 0.001**	**< 0.001**	**< 0.001**	.
	n	882	882	882	882

**Correlation is significant at the 0.01 level (2-tailed).

* Correlation is significant at the 0.05 level (2-tailed).

IMKN: Intrinsic motivation to know

EM-ID: Extrinsic motivation identification

AMO: Amotivation

We found a significant positive correlation between age and AMO; a significant negative correlation between age and IMKN; a significant negative correlation between age and EM-ID; a significant positive correlation between IMKN and EM-ID, a significant negative correlation between IMKN and AMO; a significant negative correlation between EM-ID and AMO (Table 9).

## Discussion

Our study found that the mean of independent learning style was the highest among the learning style dimensions. We think that the high level of independent learning style among our students is due to the education system in higher secondary. For this reason, it may be more appropriate for educators to provide training by taking this into consideration, at least for first graders. Unlike the results of our study, a study conducted with 545 students in Malaysia indicated that they were dominant in collaborative and competitive learning styles [15]. Another study conducted with 651 s-year university students, who received vocational education, found that competitive and cooperative learning styles were predominantly preferred by the students [16]. A study on the learning styles of physiotherapy students reported that the most common learning style was collaborative [4]. Another study conducted with 170 final year medical students from Gazi University found that they had collaborative and competitive learning styles [17]. Why the independent learning style was higher in our study may be due to the density of first-year students and may be seen as inconsistent since other studies were only conducted in senior classes. The tendency of students to work individually in high school may also be associated with a high independent learning style. However, understanding students’ learning style preferences is important for designing instruction.

Our study demonstrated that there are gender differences in learning styles. In our study males had a significantly higher mean of independent learning and avoidant learning than females (p < 0.05), and females had significantly higher means of dependent learning and participant learning than males (p < 0.05). Similarly, in the study by Amir and Jelas [15], male students had higher means of independent and avoidant learning than females, and female students had higher means of collaborative, dependent, competitive, and participative learning [15]. This shows similar results with our study. Another study on the learning styles of students reported that the dominant style of students according to gender shows that the dominant styles of male students include: cooperative, competitive and dependent while dominant styles of female students include: competitive, cooperative and dependence. More than half of the students’ dominant style was participative. In the study, more than half of male and female students preferred the participation style [18]. A study conducted with undergraduate and graduate students (n = 1039) at Tehran University found that females obtained significantly higher means in collaborative, participative, and dependent styles than males, while males had higher means than females in avoidant and independent styles [19]. Another study found that there was a significant difference between male and female students in learning styles, including participatory, avoiding, and independent. [20]. In our study, it is thought that the independent learning style being the highest learning style may be due to the students’ tendency to work individually during their education years before starting university. A study conducted to evaluate the effect of learning styles and study behaviors on preclinical medical students’ pharmacology exam scores (n = 87) reported that collaborative and competitive dominant learning styles were frequent in the cohort [21]. These findings support data from other studies conducted in Malaysia (545 medical students) [15] and Pakistan [22]. Differently in our study, we found that the mean of independent learning style was the highest among the learning style dimensions. It is thought that the independent learning style being the most common learning style in our study is may be due to the cultural differences. In our study females had significantly higher means of dependent learning and participant learning than males. The reason why female students show more participatory learning style characteristics than males may be due to their greater willingness to learn and liking to share their knowledge and ideas.

A study was conducted to examine the awareness of students (n = 372) in teaching programs of the Faculty of Education about their learning styles and self-regulation skills and to what extent they consider these learning styles and self-regulation skills in their teaching lives. It found that the “dependent learning style” was the most preferred one. It also reported that learning styles differed according to gender; females had more dependent and participatory learning styles than males and males preferred the collaborative learning style. In terms of years, the 1st year students had more dependent, collaborative, and participatory learning styles compared to the 4th year students. The analysis of the interviews with students indicated that the learning style most adopted by students was the “dependent learning style” [23]. Similarly, in our study, the cooperative learning style of the 1st grade students is higher than the 3rd grade, also the participant learning averages of the 1st grade students are higher than the 3rd -year and 5th -year students, the dependent learning averages of the 1st grade students are higher than the 5th grades and avoidant learning averages of the 1st grade students are lower than the 3rd -year and 5th -year students. The high level of dependent learning style in first grade students may be due to the fact that students go through the preparation stages for the university placement exam before they start university. During high school years, students are expected to follow the path drawn for them. Therefore, it is difficult for students to develop their autonomy skills. This situation may have caused the 1st grade students to see the instructor and resources as a source of authority and support, and to show dependent learning style characteristics. The high level of cooperative learning style among first-year students may be due to the fact that students are new to university and do not know the features of the system, they need help from other individuals, they cannot work well on their own, and they prefer to be guided by other members of the group.

Studies concerning with academic motivation, which is the other dimension of our study, when evaluated, a study conducted with medical students (n = 531) in Indonesia found that students’ the mean score of intrinsic motivation were higher than the extrinsic motivation with no differences among three groups of students [24]. A study conducted with 2nd -year medical students found that spending time with family was positively associated with IM scores [25]. In parallel with this finding, our results indicated that the students whose fathers helped while studying had significantly higher means of IMAT than those who did not receive help from their fathers.

A study examining college students’ academic motivation reported that female students had higher levels of overall motivation as well as IM and EM. It found that both IM and EM declined with years in college. Self-funded students had lower academic motivation in general and EM in particular. The relatively weak motivation of self-funded students may be because they would not have the external motivators [26]. In our study, there was no significant difference in academic motivation dimensions according to working in an income-generating job. A study conducted by Aung et al. [27] found that the students’ IM level was lower than their EM level, and IM level was not statistically different between male and female students [27]. Similarly, in our study, it was found that students working in a job had low IMKN and high amotivation. The reason of this may be related to the students’ not having enough time to meet their curiosity and desire to learn because of their work. Therefore, their intrinsic motivation may be low. Their lack of motivation can be caused by stress and physical exhaustion. In our study, males had significantly higher means of AMO than females (p < 0.05) A study conducted with 1st -year medical students (n = 138) found that EM was positively associated with being female. IM was correlated with perceived family support. AMO was related to being male [28]. Similar to our results, Brouse et al. [26] reported that females scored higher than males in all IM measurements, while the AMO scores were significantly higher in males than females. Another study by Kusurkar et al. [29] showed that females had higher strength of motivation (EM + IM) [29]. In the study by Sobral [30], male students had higher ER and AMO scores [30].

Our study found that there was no significant difference between IMAT, IMES, EM-IJ, and EM-ER by year (p > 0.05). We found that the means of AMO differed significantly according to the year (p < 0.05). Different from our results, in a study examining college students’ academic motivation reported that IM and EM declined with years [26]. This extends/agrees with /disagrees with Stover et al. [31] who when comparing the academic motivation of high school students and college students found that high school students had higher EM and AMO mean scores than college students [31].

In our study we found that there were significant relationships between independent learning and IM, between avoidant learning and EM, and between collaborative learning and IMKN, IMAT, and IMES. Similarly, in a study relationship between learning styles and motivation for higher education revealed significant relationship [7]. With the relationship between learning styles and academic motivation, we can identify students’ academic weaknesses. By providing them with habits to change these weaknesses we may increase their academic motivation.

### Limitations of the study

There were some limitations of this study. Among the limitations of the study, this study was cross-sectional. The conclusions of this study could be limited due to the cross-sectional design. Learning style and academic motivation can change based on experience and the demands of a situation. The study was conducted in only a single medical school and may therefore not be generalizable to other medical schools with students of different cultures and schools that use different academic methods.

## Conclusion

We found that there were significant relationships between independent learning and IM, between avoidant learning and EM, and between collaborative learning and IMKN, IMAT, and IMES.

In addition, we think that different teaching strategies and assessment methods can be applied to strengthen collaborative learning, participant learning, and this improvement in learning styles will lead to increase in academic motivation. We hope that this research will contribute to medical education on the subject of establishing appropriate teaching methods.

Teachers have to plan and implement activities based on students’ learning styles and academic motivation to encourage students to effectively participate in the classroom. It is necessary for students to be able to determine their learning styles to increase their own intrinsic motivation. In higher education institutions, seminars should be given to students about different learning styles and how to choose the most suitable learning style.

The results of this study can provide useful information for improving the teaching and learning process of teachers and students. However, more research needs to be undertaken to understand the relationship between learning style preferences and academic motivation in teaching and learning process.

## Electronic supplementary material

Below is the link to the electronic supplementary material.

Supplementary Material 1

## Data Availability

The datasets generated and/or analyzed during the current study are available from the corresponding author; on reasonable request.

## Abbreviations

IMKN: Intrinsic motivation to know
IMAT: Intrinsic motivation to accomplish things
IMES: Intrinsic motivation to experience stimulation
EM-ID: Extrinsic motivation identification
EM-IJ: Extrinsic motivation introjection
EM-ER: Extrinsic motivation external regulation
AMO: Amotivation
