# Supplementary material for: Learning styles of medical students and related factors
Source: BMC Med Educ. 2023 Apr 25;23:282. doi: 10.1186/s12909-023-04267-4 (PMC10131376; doi:10.1186/s12909-023-04267-4)
Supplement: Supplementary file 1 — Supplementary Material 1 [file 12909_2023_4267_MOESM1_ESM.docx]

| **Supplementary file**  **Table: Relationship between age, learning styles and academic motivation dimensions** | | | | | | | | | | | | | |
| --- | --- | --- | --- | --- | --- | --- | --- | --- | --- | --- | --- | --- | --- |
|  | | Age | Independent Learning | Avoidant Learning | Collaborative Learning | Dependent Learning | Competetive Learning | Participant Learning | IMAT | IMES | EM-IJ | EM-ER |  |
| Age | r | 1 | -0.05 | 0.20^**^ | -0.19^**^ | -0.05 | -0.09^**^ | -0.16^**^ | -0.10^**^ | -0.02 | 0.01 | 0.02 |  |
|  | p | . | 0.10 | <0.00 | <0.00 | 0.13 | 0.00 | <0.00 | 0.00 | 0.45 | 0.64 | 0.46 |  |
|  | p adjusted | . | 1.00 | **<0.00** | **<0.00** | 1.00 | **0.03** | **<0.00** | **0.01** | 1.00 | 1.00 | 1.00 |  |
|  | n | 882 | 882 | 882 | 882 | 882 | 882 | 882 | 882 | 882 | 882 | 882 |  |
| Independent Learning | r | -0.05 | 1 | 0.05 | 0.31^**^ | -0.08^*^ | 0.14^**^ | 0.10^**^ | 0.35^**^ | 0.23^**^ | 0.06 | 0.03 |  |
|  | p | 0.10 | . | 0.13 | <0.00 | 0.01 | <0.00 | 0.00 | <0.00 | <0.00 | 0.05 | 0.37 |  |
|  | p adjusted | 1.00 | . | 0.68 | **<0.00** | 0.09 | **<0.00** | **0.00** | **<0.00** | **<0.00** | 0.54 | 1.00 |  |
|  | n | 882 | 882 | 882 | 882 | 882 | 882 | 882 | 882 | 882 | 882 | 882 |  |
| Avoidant Learning | r | 0.20^**^ | 0.05 | 1 | -0.12^**^ | -0.04 | -0.04 | -0.44^**^ | -0.18^**^ | -0.11^**^ | 0.13^**^ | 0.20^**^ |  |
|  | p | <0.00 | 0.13 | . | <0.00 | 0.14 | 0.21 | <0.00 | <0.00 | 0.00 | <0.00 | <0.00 |  |
|  | p adjusted | **<0.00** | 0.68 | . | **<0.00** | 0.73 | 1.00 | **<0.00** | **<0.00** | **0.01** | **<0.00** | **<0.00** |  |
|  | n | 882 | 882 | 882 | 882 | 882 | 882 | 882 | 882 | 882 | 882 | 882 |  |
| Collaborative Learning | r | -0.19^**^ | 0.31^**^ | -0.12^**^ | 1 | 0.05 | 0.30^**^ | 0.28^**^ | 0.42^**^ | 0.25^**^ | 0.17^**^ | -0.02 |  |
|  | p | <0.00 | <0.00 | <0.00 | . | 0.10 | <0.00 | <0.00 | <0.00 | <0.00 | <0.00 | 0.48 |  |
|  | p adjusted | **<0.00** | **<0.00** | **<0.00** | . | 0.53 | **<0.00** | **<0.00** | **<0.00** | **<0.00** | **<0.00** | 1.00 |  |
|  | n | 882 | 882 | 882 | 882 | 882 | 882 | 882 | 882 | 882 | 882 | 882 |  |
| Dependent Learning | r | -0.05 | -0.08^*^ | -0.04 | 0.05 | 1 | 0.22^**^ | 0.45^**^ | -0.10^**^ | 0.17^**^ | 0.20^**^ | 0.28^**^ |  |
|  | p | 0.13 | 0.01 | 0.14 | 0.10 | . | <0.00 | <0.00 | 0.00 | <0.00 | <0.00 | <0.00 |  |
|  | p adjusted | 1.00 | 0.09 | 0.73 | 0.53 | . | **<0.00** | **<0.00** | **0.02** | **<0.00** | **<0.00** | **<0.00** |  |
|  | n | 882 | 882 | 882 | 882 | 882 | 882 | 882 | 882 | 882 | 882 | 882 |  |
| Competetive Learning | r | -0.09^**^ | 0.14^**^ | -0.04 | 0.30^**^ | 0.22^**^ | 1 | 0.41^**^ | 0.31^**^ | 0.24^**^ | 0.41^**^ | 0.14^**^ |  |
|  | p | 0.00 | <0.00 | 0.21 | <0.00 | <0.00 | . | <0.00 | <0.00 | <0.00 | <0.00 | <0.00 |  |
|  | p adjusted | **0.03** | **<0.00** | 1.00 | **<0.00** | **<0.00** | . | **<0.00** | **<0.00** | **<0.00** | **<0.00** | **<0.00** |  |
|  | n | 882 | 882 | 882 | 882 | 882 | 882 | 882 | 882 | 882 | 882 | 882 |  |
| **Table: Relationship between age, learning styles and academic motivation dimensions (Continued)** | | | | | | | | | | | | |  |
|  |  | Age | Independent Learning | Avoidant Learning | Collaborative Learning | Dependent Learning | Competetive Learning | Participant Learning | IMAT | IMES | EM-IJ | EM-ER |  |
| Participant Learning | r | -0.16^**^ | 0.10^**^ | -0.44^**^ | 0.28^**^ | 0.45^**^ | 0.41^**^ | 1 | 0.25^**^ | 0.33^**^ | 0.21^**^ | 0.05 |  |
|  | p | <0.00 | 0.00 | <0.00 | <0.00 | <0.00 | <0.00 | . | <0.00 | <0.00 | <0.00 | 0.08 |  |
|  | p adjusted | **<0.00** | **0.00** | **<0.00** | **<0.00** | **<0.00** | **<0.00** | . | **<0.00** | **<0.00** | **<0.00** | 0.82 |  |
|  | n | 882 | 882 | 882 | 882 | 882 | 882 | 882 | 882 | 882 | 882 | 882 |  |
| IMAT | r | -0.10^**^ | 0.35^**^ | -0.18^**^ | 0.42^**^ | -0.10^**^ | 0.31^**^ | 0.25^**^ | 1 | 0.57^**^ | 0.47^**^ | 0.05 |  |
|  | p | 0.00 | <0.00 | <0.00 | <0.00 | 0.00 | <0.00 | <0.00 | . | <0.00 | <0.00 | 0.13 |  |
|  | p adjusted | **0.01** | **<0.00** | **<0.00** | **<0.00** | **0.01** | **<0.00** | **<0.00** | . | **<0.00** | **<0.00** | 1.00 |  |
|  | n | 882 | 882 | 882 | 882 | 882 | 882 | 882 | 882 | 882 | 882 | 882 |  |
| IMES | r | -0.02 | 0.23^**^ | -0.11^**^ | 0.25^**^ | 0.17^**^ | 0.24^**^ | 0.33^**^ | 0.57^**^ | 1 | 0.39^**^ | 0.09^**^ |  |
|  | p | 0.45 | <0.00 | 0.00 | <0.00 | <0.00 | <0.00 | <0.00 | <0.00 | . | <0.00 | 0.00 |  |
|  | p adjusted | 1.00 | **<0.00** | **0.00** | **<0.00** | **<0.00** | **<0.00** | **<0.00** | **<0.00** | . | **<0.00** | **0.03** |  |
|  | n | 882 | 882 | 882 | 882 | 882 | 882 | 882 | 882 | 882 | 882 | 882 |  |
| EM-IJ | r | 0.01 | 0.06 | 0.13^**^ | 0.17^**^ | 0.20^**^ | 0.41^**^ | 0.21^**^ | 0.47^**^ | 0.39^**^ | 1 | 0.41^**^ |  |
|  | p | 0.64 | 0.05 | <0.00 | <0.00 | <0.00 | <0.00 | <0.00 | <0.00 | <0.00 | . | <0.00 |  |
|  | p adjusted | 1.00 | 0.48 | **<0.00** | **<0.00** | **<0.00** | **<0.00** | **<0.00** | **<0.00** | **<0.00** | . | **<0.00** |  |
|  | n | 882 | 882 | 882 | 882 | 882 | 882 | 882 | 882 | 882 | 882 | 882 |  |
| EM-ER | r | 0.02 | 0.03 | 0.20^**^ | -0.02 | 0.28^**^ | 0.14^**^ | 0.05 | 0.05 | 0.09^**^ | 0.41^**^ | 1 |  |
|  | p | 0.46 | 0.37 | <0.00 | 0.48 | <0.00 | <0.00 | 0.08 | 0.13 | 0.00 | <0.00 |  |  |
|  | p adjusted | 1.00 | 1.00 | **<0.00** | 1.00 | **<0.00** | **<0.00** | 0.73 | 1.00 | **0.03** | **<0.00** | . |  |
|  | n | 882 | 882 | 882 | 882 | 882 | 882 | 882 | 882 | 882 | 882 | 882 |  |
| **. Correlation is significant at the 0.01 level (2-tailed).  *. Correlation is significant at the 0.05 level (2-tailed). | | | | | | | | | | | | |  |

IMAT: Intrinsic motivation to accomplish things

IMES: Intrinsic motivation to experience stimulation

EM-IJ: Extrinsic motivation introjection

EM-ER: Extrinsic motivation external regulation
